# A Case of Transthyretin Cardiac Amyloidosis Coexisting With Rheumatoid Arthritis

**DOI:** 10.7759/cureus.75443

**Published:** 2024-12-10

**Authors:** Yongdeok B Shin, Jenna McAllister

**Affiliations:** 1 Graduate Medical Education (GME) Internal Medicine, Mary Washington Healthcare, Fredericksburg, USA

**Keywords:** pyrophosphate scanning, rheumatoid arthritis, transthoracic echocardiography, transthyretin cardiac amyloidosis, ttr stabilizer

## Abstract

Cardiac amyloidosis is a rare but increasingly recognized cause of heart failure, often underdiagnosed until later stages of the disease. This report describes a case of transthyretin amyloidosis (ATTR) in a 68-year-old male patient with a significant medical history of rheumatoid arthritis (RA), a combination seldom documented in the literature. The patient presented with progressive symptoms of heart failure, and diagnostic testing confirmed ATTR cardiac amyloidosis through pyrophosphate (PYP) scanning. This case highlights the clinical presentation, diagnostic process, and management of cardiac amyloidosis while exploring a potential link between RA and amyloid deposition. Early recognition of cardiac amyloidosis is crucial for improving outcomes, especially in patients with coexisting systemic inflammatory conditions like RA.

## Introduction

Amyloidosis is a systemic infiltrative disease characterized by the extracellular deposition of insoluble fibrillar proteins, leading to organ dysfunction. Cardiac amyloidosis, in particular, results from amyloid deposition in the myocardium, causing restrictive cardiomyopathy, diastolic dysfunction, and ultimately heart failure [1]. Several subtypes of amyloidosis exist, such as light-chain amyloidosis, serum amyloid A protein, transthyretin (TTR) amyloidosis, beta-2 microglobulin amyloidosis, and apolipoprotein A1 or A2 amyloidosis. Among these, TTR-related amyloidosis (ATTR) has gained increasing recognition due to its prevalence in the elderly, especially in the male population aged over 65 years. ATTR includes both hereditary and wild-type forms, with recent advancements in treatments, such as TTR stabilizers (e.g., tafamidis) and RNA-based therapies, that significantly improve patient outcomes [2]. These therapies slow disease progression, reduce hospitalizations, and enhance survival, marking a transformative shift in the management of this previously untreatable condition [1].

TTR is a transport protein primarily produced in the liver, responsible for carrying thyroxine and retinol-binding protein. In ATTR, TTR misfolds and forms amyloid fibrils that deposit in the myocardium and other tissues, resulting in progressive organ dysfunction. ATTR exists in two forms: wild-type (previously referred to as senile systemic amyloidosis) and hereditary (associated with specific TTR gene mutations) [2].

Rheumatoid arthritis (RA), a chronic systemic autoimmune disease, has been linked to various cardiovascular complications, primarily due to chronic inflammation [3]. Amyloidosis, particularly the AA subtype, is a recognized complication of RA, but its association with ATTR has not been widely studied. This case presents a unique clinical scenario where a patient with long-standing RA developed ATTR cardiac amyloidosis, raising the question of whether systemic inflammation in RA could play a role in the pathogenesis of ATTR.

## Case presentation

A 68-year-old male patient presented to the cardiology clinic with progressive dyspnea on exertion, fatigue, and lower extremity edema over the past six months. He had a 20-year history of RA, managed with leflunomide and etanercept for the past five years. Amyloidosis can be influenced by various therapies. In this case, the patient's treatment history over the first 15 years, including the use of nonsteroidal anti-inflammatory drugs (NSAIDs), disease-modifying antirheumatic drugs (DMARDs), immunomodulators, and gold therapy, may have contributed to the disease process. He had no prior history of ischemic heart disease or significant hypertension, and his RA had been well-controlled with his current regimen.

Physical examination

Upon arrival at the emergency department, the patient’s vital signs were notable for a temperature of 95.7 °F, pulse rate of 75 beats per minute, respiratory rate of 16 breaths per minute, blood pressure of 161/108 mmHg, and oxygen saturation of 96% on room air. On physical examination, the patient was noted to have elevated jugular venous pressure, peripheral pitting edema in both legs, and ascites. Cardiac auscultation revealed distant heart sounds without a discernible murmur. The lungs were clear to auscultation, but mild bilateral basal crackling was present, suggestive of early pulmonary congestion.

Investigations

Laboratory tests showed an elevated N-terminal pro-brain natriuretic peptide (NT-proBNP) level of 6,400 pg/mL and a mildly elevated troponin I level of 0.16 ng/mL, indicating myocardial strain and possible heart failure (Table 1). Renal and liver function tests were within normal limits (Table 1).

**Table 1:** Laboratory test results showing elevated N-terminal pro-brain natriuretic peptide (NT-proBNP) and troponin I

Lab Test	Results	Reference range
NT-proBNP	6,400 pg/mL	0-125 pg/mL
Troponin I	0.16 ng/mL	0-0.04 ng/mL
Hemoglobin	14.0 g/dL	13.5-17.5 g/dL (M), 12.0-15.5 g/dL (F)
Hematocrit	45%	41%-50% (M), 36%-44% (F)
White blood cell count	7,200/µL	4,000-11,000/µL
Platelet count	328,000/µL	150,000-450,000/µL
Sodium	140 mmol/L	135-145 mmol/L
Potassium	4.5 mmol/L	3.5-5.0 mmol/L
Chloride	102 mmol/L	98-106 mmol/L
Bicarbonate	24 mmol/L	22-29 mmol/L
Blood urea nitrogen (BUN)	13 mg/dL	7-20 mg/dL
Creatinine	1.1 mg/dL	0.6-1.2 mg/dL
Glucose	95 mg/dL	70-99 mg/dL (fasting)
Calcium	9.7 mg/dL	8.5-10.2 mg/dL
Total protein	7.7 g/dL	6.0-8.3 g/dL
Albumin	4.2 g/dL	3.5-5.0 g/dL
Aspartate aminotransferase (AST)	13 U/L	10-40 U/L
Alanine aminotransferase (ALT)	21 U/L	7-56 U/L
Alkaline phosphatase (ALP)	87 U/L	44-147 U/L

An electrocardiogram (ECG) revealed low-voltage QRS complexes in the limb leads and a left bundle branch block (LBBB). This finding, combined with the clinical suspicion of restrictive cardiomyopathy, raised concerns for infiltrative cardiac diseases such as amyloidosis.

**Figure 1:**
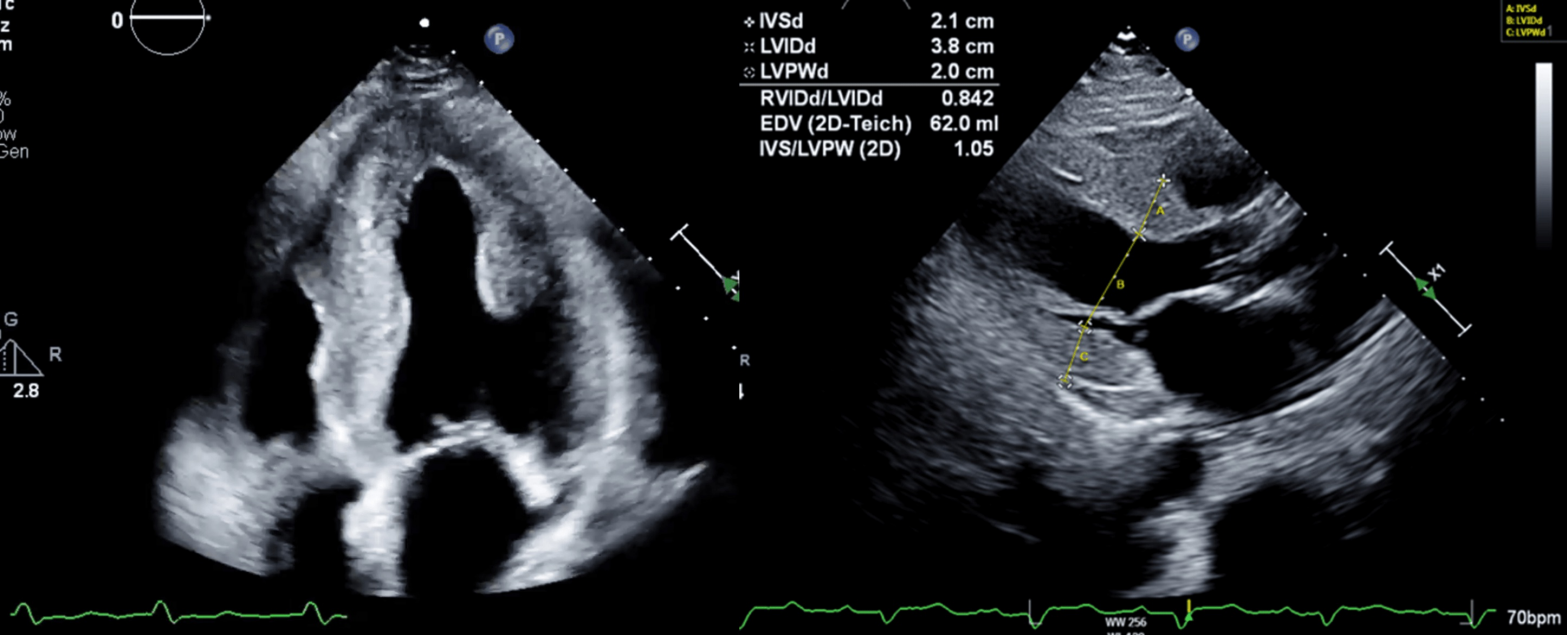
Severe concentric left ventricular hypertrophy with thickened interventricular septum measuring 2.1 cm

Transthoracic echocardiography (TTE) demonstrated severe concentric left ventricular hypertrophy with a moderately reduced ejection fraction (35%-40%). There was evidence of bi-atrial enlargement and a restrictive filling pattern on Doppler analysis, consistent with restrictive cardiomyopathy. Left ventricular wall thickness measured 16 mm, significantly thicker than expected in hypertensive cardiomyopathy, suggesting a potential infiltrative process like amyloidosis (Figure 1).

**Figure 2:**
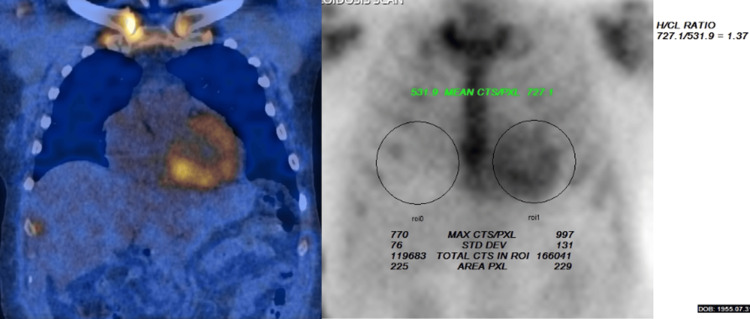
Visual semiquantitative radiotracer uptake in the myocardium from the SPECT-CT is more than rib uptake (grade 3). Quantitatively, the heart to contralateral lung ratio (H/CL) is 1.37

Given these findings, a technetium-99m pyrophosphate (PYP) scan was ordered to evaluate for cardiac amyloidosis. The PYP scan revealed a heart-to-contralateral lung (H/CL) ratio of 1.37 and a semiquantitative visual score of 2-3, both strongly suggestive of ATTR cardiac amyloidosis (Figure 2). These results confirmed the diagnosis of wild-type ATTR cardiac amyloidosis (ATTRwt), eliminating the need for an invasive endomyocardial biopsy.

## Discussion

ATTR results from the misfolding of transthyretin protein, leading to amyloid deposition within the myocardium. It has two forms: hereditary and wild-type, with the latter being more common in the elderly male population [4]. In this case, the patient’s PYP scan was diagnostic for wild-type ATTR, which typically affects individuals over the age of 65 and is associated with restrictive cardiomyopathy and progressive heart failure [4]. The heart-to-contralateral lung (H/CL) ratio on the PYP scan, combined with the semiquantitative visual score, provided a highly specific and noninvasive means of confirming the diagnosis of ATTR [4].

Cardiovascular disease (CVD) disproportionately affects racial, ethnic, and socioeconomically disadvantaged groups, perpetuating significant health inequities. Black, Hispanic, and Indigenous populations exhibit higher rates of hypertension, obesity, diabetes, and heart failure, resulting in greater CVD-related mortality [5]. Structural barriers, including implicit biases in healthcare systems, underdiagnosis, and limited access to care, exacerbate these disparities [5]. Lower socioeconomic status further compounds the risks through factors like reduced healthcare access, poor living conditions, and limited education, which hinder health literacy and disease management. Chronic stress and systemic inequities such as redlining and food insecurity amplify these challenges [5].

RA is a systemic inflammatory disease primarily affecting the joints but has been linked to various extra-articular manifestations, including CVD [2]. Chronic inflammation in RA leads to an increased risk of atherosclerosis, myocardial dysfunction, and heart failure. Traditionally, amyloidosis associated with RA has been attributed to the AA type, which results from chronic overproduction of serum amyloid A protein, an acute-phase reactant [2].

However, the role of RA in predisposing patients to other forms of amyloidosis, such as ATTR, remains underexplored. In this case, chronic systemic inflammation from RA may have contributed to the pathogenesis of ATTR amyloidosis by promoting an environment conducive to protein misfolding and amyloid fibril deposition. This hypothesis is supported by recent studies suggesting that chronic inflammation in RA can influence the development of various amyloidogenic proteins, including TTR [1,6].

Moreover, the use of DMARDs and biologics, such as leflunomide and etanercept, in patients with RA has been associated with both beneficial and adverse effects on cardiovascular health. While these agents help control inflammation, their long-term effects on cardiac amyloid deposition remain unclear [7]. Further research is needed to elucidate the potential link between RA, chronic inflammation, and the development of ATTR amyloidosis. 

The diagnosis of cardiac amyloidosis requires a high degree of clinical suspicion, particularly in elderly patients presenting with heart failure symptoms, unexplained left ventricular hypertrophy, and low-voltage QRS complexes on ECG [8]. Myocardial radiotracer uptake demonstrated a sensitivity exceeding 99% and a specificity of 86% for diagnosing cardiac transthyretin amyloidosis ATTR cardiomyopathy. False positives were almost exclusively observed in patients with cardiac AL amyloidosis [8]. Notably, when grade 2 or 3 myocardial radiotracer uptake was combined with the absence of monoclonal protein in serum or urine, the specificity and positive predictive value for cardiac ATTR amyloidosis reached 100% [8]. In this case, the combination of clinical findings and imaging studies, particularly the PYP scan, facilitated a timely diagnosis of ATTR cardiac amyloidosis without the need for invasive procedures [9].

Cardiac magnetic resonance imaging (CMR) provides significant diagnostic utility in cardiac amyloidosis, with characteristic findings such as restrictive morphology, altered gadolinium kinetics, and extracellular volume expansion detected via T1 mapping. However, false-positive and false-negative results are not uncommon, with a sensitivity of 93%, a specificity of 70%, and an overall negative predictive accuracy of 84% for cardiac amyloidosis [9]. Additionally, CMR is expensive, limited to availability at specialized centers, contraindicated in a considerable number of patients, and unable to reliably differentiate between amyloid subtypes [8]. Endomyocardial biopsy remains the gold standard for diagnosing amyloidosis but carries procedural risks, particularly in elderly patients with multiple comorbidities [8]. 

Once the diagnosis of ATTR cardiac amyloidosis was confirmed, the patient was referred for evaluation for targeted therapies [9]. Tafamidis, a TTR stabilizer, was initiated as a treatment option to halt the progression of amyloid deposition and preserve cardiac function [9]. The patient's RA management remained unchanged, as his symptoms were well-controlled with leflunomide and etanercept.

Given the progressive nature of ATTR, the patient was closely monitored for signs of worsening heart failure. Guidelines recommend angiotensin-converting enzyme (ACE) inhibitors and beta-blockers (BBs) as standard therapy for all patients with symptomatic heart failure and reduced ejection fraction, regardless of the underlying etiology [10]. However, some studies suggest that guideline-directed medical therapy provides little to no significant benefit in patients with ATTR cardiac amyloidosis, unless comorbid conditions such as coronary artery disease or hypertension are present [10]. His treatment plan included optimized heart failure therapy with BBs, ACE inhibitors, and diuretics. 

## Conclusions

This case highlights the importance of considering cardiac amyloidosis, particularly ATTR, in patients with progressive heart failure and unexplained left ventricular hypertrophy, especially in the context of systemic diseases like RA. The potential association between RA and ATTR amyloidosis underscores the need for further investigation into the role of chronic inflammation in amyloid deposition. Early recognition and appropriate management of cardiac amyloidosis can improve patient outcomes and quality of life, particularly with the advent of disease-modifying treatments for ATTR.

## References

[1] Siddiqi OK, Ruberg FL (2018). Cardiac amyloidosis: an update on pathophysiology, diagnosis, and treatment. Trends Cardiovasc Med.

[2] Tsuda R, Shinoda K, Ushijima R, Nakamura M, Katoh N, Imura J, Tobe K (2021). A case of wild-type transthyretin cardiac amyloidosis with rheumatoid arthritis. Mod Rheumatol Case Rep.

[3] Voskuyl AE (2006). The heart and cardiovascular manifestations in rheumatoid arthritis. Rheumatology (Oxford).

[4] Falk RH, Alexander KM, Liao R, Dorbala S (2016). AL (light-chain) cardiac amyloidosis: a review of diagnosis and therapy. J Am Coll Cardiol.

[5] Borkowski P, Borkowska N, Mangeshkar S, Adal BH, Singh N (2024). Racial and socioeconomic determinants of cardiovascular health: a comprehensive review. Cureus.

[6] Maurer MS, Elliott P, Comenzo R, Semigran M, Rapezzi C (2017). Addressing common questions encountered in the diagnosis and management of cardiac amyloidosis. Circulation.

[7] Aviña-Zubieta JA, Choi HK, Sadatsafavi M, Etminan M, Esdaile JM, Lacaille D (2008). Risk of cardiovascular mortality in patients with rheumatoid arthritis: a meta-analysis of observational studies. Arthritis Rheum.

[8] Gillmore JD, Maurer MS, Falk RH (2016). Nonbiopsy diagnosis of cardiac transthyretin amyloidosis. Circulation.

[9] Donnelly JP, Hanna M (2017). Cardiac amyloidosis: an update on diagnosis and treatment. Cleve Clin J Med.

[10] Aus dem Siepen F, Hein S, Hofmann E (2024). Prognostic value of standard heart failure medication in patients with cardiac transthyretin amyloidosis. J Clin Med.

